# Complete Genome Sequence of *Pseudomonas aeruginosa* Podophage MPK7, Which Requires Type IV Pili for Infection

**DOI:** 10.1128/genomeA.00744-13

**Published:** 2013-10-10

**Authors:** Hee-Won Bae, You-Hee Cho

**Affiliations:** Department of Pharmacy, College of Pharmacy, CHA University, Gyeonggi-do, South Korea

## Abstract

We report the complete genome sequence of *Pseudomonas aeruginosa* podophage MPK7. It displays synteny to the *P. aeruginosa* phages of the *Phikmvlikevirus* genus, which includes phiKMV and LKA1. MPK7 requires type IV pili (TFP) for infection, suggesting the role of functional TFP as the receptor for this phage genus.

## GENOME ANNOUNCEMENT

Acute and chronic infections caused by *Pseudomonas aeruginosa*, an opportunistic human pathogen, pose a serious threat to patients hospitalized with cancer, cystic fibrosis, and severe burns (1). The increasing emergence of multidrug resistance has reduced the number of clinically available antibiotics that retain activities against this pathogen. Thus, more effective and alternative treatment strategies for *P. aeruginosa* infections urgently need to be developed, and several studies have begun to characterize nonantibiotic approaches; recent advances in phage therapy based on large reservoirs of bacteriophages have shown relevant efficacy toward various *P. aeruginosa* infections in animal models (2).

We have recently isolated a new phage, MPK7, from local sewage samples, which forms plaques on *P. aeruginosa* strain PAO1. Based on its virion structure, it is a podophage (3). MPK7 requires functional type IV pili (TFP) for infections but displays a narrower host range than MP22, a siphophage that requires TFP for infection (4), suggesting the presence of other host specificity determining factors than the phage receptor. To elucidate the phage elements that are involved in host specificity, we determined the complete genome sequence of *P. aeruginosa* MPK7.

The genomic DNA of MPK7 was prepared as described previously (4) and sequenced using the GS FLX Titanium platform by the local service provider (Macrogen, Seoul, South Korea). The genome was assembled and analyzed using the Roche GS FLX software (version 2.6), and the potential open reading frames (ORFs) that encode proteins of >30 amino acids were determined using the GeneMark software (5). Annotation was performed using BLASTx searches against the GenBank (http://www.ncbi.nlm.nih.gov/genbank) and UniProt (http://www.uniprot.org) databases.

MPK7 has a linear 42,874-bp DNA genome with a G+C content of 62.14% and 54 ORFs in one orientation; it displays synteny to the *P. aeruginosa* podophages of the *Phikmvlikevirus* genus, such as phiKMV and LKA1. The length of the terminal repeat has not been determined. No tRNA gene has been identified in the genome using the tRNAscan-SE version 1.21 (6). The modular organization of the MPK7 genome follows the conserved pattern found in the *Phikmvlikevirus* strains: genome synthesis (putative DNA binding protein, DNA ligase, exonuclease, and endonuclease), genome expression (DNA-dependent RNA polymerase), virion assembly (head-tail connector protein, major and minor capsid proteins, internal virion proteins, tail tubular proteins, tail fiber proteins, and DNA terminase), and host lysis (holin, endolysin, and spanin proteins). The majority of the hypothetical proteins reside in the early and middle regions, indicative of their involvement in nucleotide metabolism and DNA synthesis. Recently, the products of two genes (*gp13* and *gp21*) of *P. aeruginosa* podophage LUZ19 were shown to block bacterial DNA replication (7). Based on this and the overall synteny in the genomes of *Phikmvlikevirus* phages, further elucidation of the functions of the unique hypothetical phage proteins conserved in this genus may provide new insight into the mechanisms of the genome evolution of the podophages in regards to killing efficacy and host specificity.

### Nucleotide sequence accession number.

The complete genome sequence of phage MPK7 has been deposited in GenBank under the accession no. JX501340.
